# Interleukin-1 blockade with RPH-104 in patients with acute ST-elevation myocardial infarction: study design and rationale

**DOI:** 10.1186/s12967-021-02828-z

**Published:** 2021-04-26

**Authors:** M. Samsonov, V. Bogin, B. W. Van Tassell, A. Abbate

**Affiliations:** 1 R-Pharm JSC, Moscow, Russia; 2 Cromos Pharma, LLC, Portland, OR, USA; 3 School of Pharmacy, Virginia Commonwealth University, Richmond, VA USA; 4 Pauley Heart Center, Virginia Commonwealth University, Richmond, VA USA

**Keywords:** STEMI, Myocardial infarction, Cardiovascular diseases, Inflammation, RPH-104, Trial design, IL-1

## Abstract

**Background:**

Myocardial injury of ST-segment elevation myocardial infarction (STEMI) initiates an intense inflammatory response that contributes to further damage and is a predictor of increased risk of death or heart failure (HF). Interleukin-1 (IL-1) is a key mediator of local and systemic inflammatory response to myocardial damage. We postulate that the use of the drug RPH-104, which selectively binds and inactivates both α and β isoforms of IL-1 will lead to a decrease in the severity of the inflammatory response which will be reflected by decrease in the concentration of hsCRP, as well as the rate of fatal outcomes, frequency of new cases of HF, changes in levels of brain natriuretic peptide (BNP) and changes in structural and functional echocardiographic parameters.

**Methods:**

This is a double blind, randomized, placebo-controlled study in which 102 patients with STEMI will receive a single administration of RPH-104 80 mg, RPH-104 160 mg or placebo (1:1:1). The primary endpoint will be hsCRP area under curve (AUC) from day 1 until day 14. Secondary endpoints will include hsCRP AUC from day 1 until day 28, rate of fatal outcomes, hospitalizations due to HF and other cardiac and non-cardiac reasons during 12-month follow-up period, frequency of new cases of HF, changes in levels of brain natriuretic peptide (BNP, NT-pro-BNP), changes in structural and functional echocardiographic parameters during 12-month follow-up period compared to baseline. The study started in October 2020 and is anticipated to end in 2Q 2022.

*Trial registration*: ClinicalTrials.gov, NCT04463251. Registered on July 9, 2020

## Background

Coronary Heart Disease (CHD) is the leading cause of death worldwide. In the United States approximately 18.2 million Americans ≥ 20 years of age have CHD. Based on 2017 mortality data CHD mortality was 365,914, and CHD any-mention mortality was 541,008. [1].

Acute Myocardial Infarction (AMI) is the most relevant form of coronary heart disease that is characterized by high mortality. On the basis of pooled data from the FHS, ARIC, CHS, MESA, CARDIA, and JHS studies of the NHLBI (1995–2012), within 1 year after a first MI at ≥ 45 years of age, 18% of males and 23% of females will die. [1] CHD remains the number one cause of death in the European Union. [2] ST-segment elevation myocardial infarction (STEMI) is a clinical syndrome of acute myocardial ischemia and necrosis associated with high risk of in-hospital and long-term morbidity and mortality [3]. According to the European Register, mortality among patients with STEMI during hospitalization ranges from 4 to 12%, mortality over 6 months can exceed 12% and over 5 years can reach 20% [4].

The main therapeutic measures in AMI are aimed at myocardial reperfusion as soon as possible with the restoration of blood flow by percutaneous coronary intervention (PCI) followed by guideline-directed medical therapy to prevent secondary events and progression to heart failure (HF). Myocardial injury initiates an intense inflammatory response that contributes to further damage and is a predictor of increased risk of death or HF [5–7]. HF is defined as “a clinical syndrome resulting from any structural or functional cardiac disorder that impairs the ability of the ventricle to fill or eject blood” [8]. The global economic cost of heart failure is estimated at $108 billion per year, comprising direct costs to healthcare systems and indirect costs to society through loss of productivity. The greatest expenditure is in the last 3 months of life [9]. Patients with known heart failure who need to be admitted to hospital for acute decompensation have high mortality rates; up to one in six patients die during admission or within 30 days after discharge [10].

STEMI survivors are at high risk for the development of HF during the initial hospitalization or years after the index event [11]. Despite significant success in treating STEMI, more than 20% of survivors develop HF within 1 year and CHD remains the most common cause of HF [12]. In separate studies, it was also shown that in the presence of signs of persistent inflammation (measured by increased concentration of hsCRP) and hemodynamic stress (measured by increased NT-pro-BNP), patients have the highest risk for developing HF [6, 13]. In experimental animal models of AMI due to surgical coronary artery ligation, the degree of the inflammatory response was a strong predictor of adverse cardiac remodeling independent of infarct size [14]. Similarly, in patients with AMI, the intensity of the inflammatory response, reflected in levels of circulating biomarkers, predicts adverse cardiac remodeling, HF, and death [15]. Given the aforementioned correlations, modulation of the inflammatory response represents an intriguing target for therapeutic intervention.

Interleukin-1 (IL-1) is among the most powerful inducers of innate immunity [16, 17]. It is a key mediator of local and systemic inflammatory response to myocardial damage. Preclinical studies have shown that inhibition of IL-1 improves the remodeling after the heart attack and prevents the development of heart failure [18]. Thus, interleukin‐1 (IL‐1) blockade is a favorable target for modulating myocardial inflammatory response. Studies have shown that an increased concentration of a highly sensitive C-reactive protein (hsCRP), which is a marker of the inflammatory response and a surrogate marker of IL-1 activity, in patients with acute coronary syndrome/myocardial infarction is independently associated with a risk of adverse cardiovascular outcomes in subsequent events (including HF) [6, 13, 19].

RPH-104 belongs to the class of targeted drugs acting on IL-1. IL-1 exists in 2 isoforms: IL-1β it is the main soluble form, functioning as a cytokine, released after its processing in the inflammasome [20]. IL-1α is another member of the IL-1 family, active already in its proform, and released during cell death, functioning as the key ‘alarmin’ that alerts the host to injury or damage [21]. RPH-104 is a hybrid protein that selectively binds and inactivates both circulating IL-1ß and IL-1α. It is a TRAP molecule that is small in size, has strong affinity for both IL-1 isoforms and best in class tissue penetration and protein stability [22]. Based on preclinical studies, RPH-104 is being developed for use in humans for the treatment of diseases associated with increased activity of IL-1ß.

## Methods

### Design

Our trial is a multicenter, phase IIa, double-blind, randomized, placebo-controlled clinical study comparing single administration of RPH-104 80 mg, RPH-104 160 mg and placebo (1:1:1 randomization) in subjects with STEMI at the study sites in the Russian Federation and in the USA. Potential patients will be assessed for eligibility and sign and informed consent form prior to randomization and study drug administration. The following procedures will be performed during the screening: collection of medical history, recording previous and concomitant therapy, demographic data, recording a 12-lead ECG.

### Overall objective

#### Patient inclusion and exclusion criteria



### Patient randomization and treatment allocation

Consented patients will be randomized in 1:1:1 ratio without stratification into one of treatment groups: RPH-104 80 mg, RPH-104 160 mg or placebo based on randomization scheme prepared using the relevant software by the responsible study statistician. Distribution will be made using block, non-adaptive, centralized randomization using Interactive Web Response System (IWRS).

The study will be double-blinded. Detailed description of operational peculiarities will be presented in an individual guideline on medicinal product handling. Given that administration of RPH-104 at 160 mg is only possible by two 80 mg injections (2 mL) at different sites and appearance of the finished forms of test product and placebo may differ, the following dosing regimen will be used to assure double blind design:

- RPH-104 80 mg group: Subjects will receive 2 mL (80 mg) of RPH-104 and 2 mL of placebo at different administration sites.
- RPH-104 160 mg group: Subjects will receive 2 mL (80 mg) of RPH-104 and 2 mL of (80 mg) of RPH-104 at different administration sites.
- Placebo group: Subjects will receive 2 mL of placebo and 2 mL of placebo at different administration sites.

### Outcomes

#### Efficacy outcomes

The primary endpoint will include hsCRP area under curve (AUC) from day 1 (baseline) until day 14. The secondary endpoints will include hsCRP area under curve (AUC) from day 1 (baseline) until Day 28, rate of fatal outcomes (cardiac and non-cardiac), hospitalizations (due to HF and other cardiac reasons not associated with HF or due to non-cardiac reasons), frequency of new cases of HF (defined as hospitalization due to HF or necessity in a loop diuretic administration intravenously or oral dose doubling in the relevant clinical facilities), changes in levels of brain natriuretic peptide (BNP, NT-pro-BNP) during 12-month follow-up period compared to baseline and changes in structural and functional echocardiographic parameters, including, but not limited to, left ventricular (LV) dimensions, LVMI, systolic and diastolic function after 12 months compared to baseline. An independent study outcome assessment committee (ISOAC) will be arranged to assure reliability and quality of data on assessment of cardiovascular and other protocol-defined outcomes as efficacy parameters. The committee will include three independent cardiologists with the relevant qualification for outcome classification according to terminology criteria 2017 Cardiovascular and Stroke Endpoint Definitions for Clinical Trials [23].

### Statistical analysis

#### Sample size justification

Rationale for sample size will be based on testing the hypothesis of statistical superiority to compare hsCRP area under curve AUC between day 1 and day 14 (primary endpoint) between each RPH-104 group and placebo. Given exploratory nature of the study, adjustment of α-level due to multiple comparisons (two RPH-104 doses and placebo) will not be used.

Assuming that the expected mean hsCRP AUC at 14 days will be 350 ± 250 mg/L for the subjects with STEMI in placebo group and standardized effect size (d Cohan) 0.80 for the lowest dose (conservative estimate based on 34 randomized subjects in each treatment group (1:1:1) (102 randomized subjects in total) will assure the study power > 90% for comparison of lower dose with placebo and study power > 95% to detect the expected further hsCRP AUC 50% reduction by 50% and increased effect of the higher RPH-104 dose compared to placebo. Unadjusted P values will be reported throughout, with statistical significance set at the 2‐tailed 0.025 levels for the primary analysis, to adjust for multiplicity. Given proportion of withdrawals and/or 20% statistical analysis (conservative estimate), study power > 80% will be maintained for all comparisons. Given potential screening failures of up to 30%, the study will enroll 146 subjects with the intent to randomize 102 subjects.

#### Primary outcome analysis

The following hypotheses will be tested for each RPH-104 dose level:

Null hypothesis H0: difference between mean AUC1-14 days CRP in RPH-104 group and mean AUC1-14 days CRP in placebo group is equal to 0.

Two-sided alternative hypothesis H1: difference between mean AUC1-14 days CRP in RPH- 104 group and mean AUC1-14 days CRP in placebo group is different from 0.

Individual AUC1-14 days CRP values will be calculated using trapezoidal method. Mean AUC1-14 days CRP value will be compared between RPH-104 and placebo groups using analysis of variance ANOVA. Mean AUC values for RPH-104 80 mg, RPH-104 160 mg and placebo groups, differences in mean values and relevant two-sided 95% confidence intervals and well as p-values will be presented.

#### Secondary outcome analysis

Analysis will be done similar to the analysis of the primary endpoint described above.

- Rate of fatal outcomes (cardiac and non-cardiac), hospitalizations (due to HF and other cardiac reasons not associated with HF or due to non-cardiac reasons) during 12-month follow-up period.
- Frequency of new cases of HF (defined as hospitalization due to HF or necessity in a loop diuretic administration intravenously or oral dose doubling in the relevant clinical facilities) during 12-month follow-up period.
  Time to event parameters will be analyzed using survival analyses (including Kaplan–Meier estimate) and compared using log-rank test if applicable. The number and proportions of subjects (of the number of FAS subjects and valid  %) with fatal outcomes, subjects with hospitalizations and subjects with new cases of HF will be presented by treatment groups. Intergroup comparisons will also be performed using exact Fisher’s test.
- Changes in levels of brain natriuretic peptide (BNP, NT-pro-BNP) during 12-month follow-up period compared to baseline.
  Changes in BNP, NT-pro-BNP levels will be presented by descriptive statistics by the study visits and treatment groups. Changes relative to baseline will be calculated for each visit. To test statistical significance of changes post baseline in the treatment groups, paired Student’s test will be used (in case of major deviations from normal law of distribution ln-transformation or nonparametric methods will be used if applicable).
  Intergroup comparisons of mean changes in BNP, NT-pro-BNP by visits will be made using analysis of covariance (ANCOVA) including baseline as a covariate and treatment group as a factor.
- Changes in structural and functional echocardiographic parameters including, but not limited, to, left ventricular (LV) dimensions, LVMMI, systolic and diastolic function after 12 months compared to baseline.

The parameters will be analyzed similar to the analysis of changes in brain natriuretic peptide level described above.

If the assumptions underlying analysis of variance (ANOVA)/analysis of covariance (ANCOVA), are violated, ln-transformation, rank ANOVA/ANCOVA or non-parametric methods will be used if applicable. Where ln-transformation is used, the relevant descriptive statistics will also include geometric mean value.

In addition, changes in marker and CRP values (based on the original scheme or after transformation) will be analyzed using mixed model repeated measures (MMRM), if applicable.

#### Safety analysis

Safety analysis will be carried out on safety set. Scope of application will be presented specifying the following variables: the number of subjects receiving RPH-104 80 mg, the number of subjects receiving RPH-104 160 mg and the number of subjects receiving placebo. Adverse events will be coded using Medical Dictionary for Regulatory Activities (MedDRA). The number and percentage of the subjects with AE/SAE, overall number and percentage of recorded AE/SAE, the number and percentage of AE/SAE resulting in early withdrawal will be presented by system organ class, preferred terms and treatment groups. The data will also be generalized by the number and percentage of AEs/SAEs with various categories of causality, expectedness and severity. All AEs will be additionally presented as lists. Quantitative safety laboratory parameters will include the measurement of a complete blood count with differential, complete metabolic panel, CPK and CK-MB, collected at days 1,14, and 28 and the results will be presented using descriptive statistics by visits and treatment groups. Changes relative to baseline and abnormal laboratory values will also be provided. The data on the number of subjects with abnormal laboratory values will be generalized for the whole study period, by visits and treatment groups. All laboratory findings will be presented as lists. Vital signs will be presented using descriptive statistics by visits and treatment groups. Changes relative to baseline will also be presented.

## Discussion

IL‐1 is a key inflammatory cytokine involved in virtually every inflammatory response and plays a critical role in the pathophysiologic sequelae of AMI [5]. In experimental mouse models of AMI due to surgical coronary artery ligation, genetic deletion of the IL‐1 type 1 receptor (IL‐1R1) protects against adverse cardiac remodeling, whereas genetic deletion of the naturally occurring receptor antagonist (IL‐1 receptor antagonist [IL‐1Ra]) amplifies the response to IL‐1 and promotes worse cardiac remodeling compared with wild‐type mice [24].

We propose to measure the area-under-the-curve for C-reactive protein (CRP) as the preferred pro-inflammatory marker in cardiovascular disease. CRP is indeed a strong predictor of adverse outcomes in STEMI. Serum levels of IL-1α and IL-1β are generally very low, often undetectable, and their predictive values have not validated in large scale studies as CRP. Interleukin-6, is a secondary cytokine downstream of IL-1 that induces CRP production in the liver. IL-6 levels correlate closely with CRP levels, and generally add little on top of CRP levels.

Rilonacept (Arcalyst; Kiniksa Pharmaceuticals, London, UK) is an IL-1 blocker approved for treatment of Cryopyrin-Associated Periodic Syndromes (CAPS) and of recurrent pericarditis. While RPH-104 and Rilonacept are both the drugs based on ‘Trap’ technology, that fuses two receptor components and a portion of an antibody’s ‘Fc’ region and while they appear to have similar affinity to IL-1 receptor, RPH-104 and Rilonacept are two distinct molecules. RPH-104 has a significantly smaller molecular mass (~ 150 kDa vs. ~ 250 kDa), that may facilitate tissue penetration. Additionally, it has an efficient heterodimer assembly with “knob- into-hole” design of Fc fragments that is devoid of Rilonacept’s homodimer formation. And lastly, RPH-104’s simpler manufacturing process makes RPH-104 significantly less costly to produce that Rilonacept.

Canakinumab (Ilaris; Novartis, Bazel, Switzerland) is a human monoclonal antibody targeted at IL-1β. Randomized, double-blind, placebo-controlled study of canakinumab (CANTOS) included 10,061 patients with myocardial infarction and an hsCRP concentration of 2 mg/L or higher. Patients received 50 mg, 150 mg, or 300 mg of canakinumab or placebo subcutaneously every 3 months. It was shown that after a single injection of the drug in patients who achieved a decrease in hsCRP below 2 mg/L, there was a decrease in the frequency of serious adverse cardiovascular events by 25% and mortality by 31% compared with patients who did not achieve a decrease in hsCRP concentration. Similar results were shown for other outcomes including hospitalization due to unstable angina pectoris requiring unplanned revascularization [26, 27].

Anakinra, a recombinant human IL‐1Ra (Kineret; Biovitrum, Stockholm, Sweden) is approved for the treatment of rheumatoid arthritis and is generally well tolerated following daily subcutaneous injection [25]. Mice treated with daily injections of anakinra had improved survival at 7 days after large anterior AMI, and the survivors had evidence of more favorable cardiac remodeling (smaller left ventricular [LV] end‐diastolic and end‐systolic diameters), higher LV ejection fraction, and reduced cardiomyocyte apoptosis [24].

Based on the preliminary benefits observed in the experimental AMI model and the established safety profile of anakinra, 2 pilot clinical trials were conducted with anakinra in ST‐segment elevation myocardial infarction (STEMI): VCUART [8] and VCUART2 [28]. Collectively, these phase 2 pilot studies enrolled 40 patients with reperfused STEMI and randomized them (within 12 h of coronary angiography) to daily treatment with anakinra 100 mg or placebo for 14 days. Anakinra was well tolerated and reduced serum levels of C‐reactive protein (CRP), a surrogate marker of IL‐1 activity. The benefits of anakinra on the incidence of HF persisted at mid‐ and long‐term follow‐up [28]. VCUART3 compared anakinra given once daily (standard dose) or twice daily (high dose) versus placebo in patients with STEMI, measuring the effects on acute inflammatory response as primary endpoint [29]. All 99 patients were enrolled within 12 h of presentation. The primary outcome was the area under the curve for C-reactive protein levels (CRP-AUC) using a high-sensitivity assay at 14 days. Two pre-specified exploratory clinical efficacy endpoints were assessed at 1 year included: 1. Composite endpoint of all-cause death or incidence of new onset HF (defined as new-onset HF requiring hospitalization, IV diuretic use in outpatient setting or a new prescription of a loop diuretic) and 2. Composite endpoint of all-cause death or hospitalization for HF. IL-1 blockade with Anakinra was well tolerated with no treatment related serious adverse events in patients with STEMI. Anakinra significantly reduced the systemic inflammatory response compared with placebo which was manifested by a significantly lower CRP AuC [29]. Prespecified exploratory analyses on clinical endpoints demonstrated a reduced incidence of HF and reduced HF hospitalizations, supporting the potential clinical benefit of IL-1 blockade in patients with acute myocardial infarction [29].

Given the similar mechanism of action of RPH-104 and anakinra, it is expected that the use of the drug RPH-104 will lead to a decrease in the severity of the inflammatory response in case of myocardial damage in patients with AMI and thus to a decrease in the concentration of hsCRP, as well as to an improvement in short-term and long-term cardiovascular outcomes, and specifically a reduction in the development of HF.

While RPH-104 and anakinra have certain similarities in their MOAs, in vitro studies of RPH-104 show that it has a significantly higher affinity to both proinflammatory isoforms of IL-1. The same in vitro studies have demonstrated a significantly higher potency of RPH-104. RPH-104 has also a significantly longer half-life [22]. Thus, it is expected that the use of a single administration of the RPH-104 will lead to inhibition of the inflammatory response in case of myocardial damage in patients with AMI as reflected by a reduced concentration of hsCRP, as well as to an improvement in short-term and long-term cardiovascular outcomes, and specifically a reduction in the development of HF. The simplicity of a single administration will improve both healthcare provider and patient acceptance of the drug, and account for 100% compliance. And lastly, the infrequency of injection site reactions will likely be viewed as, albeit small, but welcome differentiator.

Moreover, given that anakinra is yet to undergo pivotal trials to demonstrate its efficacy, and because RPH-104 may have certain advantages over other drugs in its class, our study may have a significant impact on the direction of future research into modulation of myocardial inflammatory response.

Colchicine has been recently shown to reduce the rate of recurrent cardiovascular events in patients with atherosclerotic disease [30, 31]. This is of interest because, colchicine acts as microtubule function inhibitor and interferes also with the formation and function of the inflammasome thus reducing the production and release of IL-1β [18]. At difference with the proposed study, colchicine was not used to blunt the acute inflammatory response, it was not initiated acutely during the ischemic insult, and it was used to reduce recurrent atherothrombotic events over time. As such, the scope of the colchicine studies is substantially different, and more resembling of those of CANTOS trial with canakinumab [26, 27]. Of note, while colchicine is a very useful anti-inflammatory drug, IL-1 blockers are often used in patients with severe gout or pericarditis when colchicine is insufficient, and as such IL-1 blockers are considered superior to colchicine as anti-inflammatory treatments.

In conclusion, the proposed study will determine whether IL-1 blockade using a ‘trap’ pharmacology approach with RPH-104 inhibiting both IL-1α and IL-1β can inhibit the systemic inflammatory as measured by CRP serum levels and result in favorable safety and efficacy secondary outcomes.

## Data Availability

Unrestricted.
